# Chimeric vaccine design against the conserved TonB-dependent receptor-like β-barrel domain from the outer membrane tbpA and hpuB proteins of *Kingella kingae* ATCC 23330

**DOI:** 10.3389/fmolb.2023.1258834

**Published:** 2023-11-20

**Authors:** Mutaib M. Mashraqi, Ahmad Alzamami, Norah A. Alturki, Hassan H. Almasaudi, Ibrar Ahmed, Saleh Alshamrani, Zarrin Basharat

**Affiliations:** ^1^ Department of Clinical Laboratory Sciences, College of Applied Medical Sciences, Najran University, Najran, Saudi Arabia; ^2^ Clinical Laboratory Science Department, College of Applied Medical Science, Shaqra University, AlQuwayiyah, Saudi Arabia; ^3^ Clinical Laboratory Science Department, College of Applied Medical Science, King Saud University, Riyadh, Saudi Arabia; ^4^ Alpha Genomics Private Limited, Islamabad, Pakistan; ^5^ Group for Biometrology, Korea Research Institute of Standards and Science (KRISS), Daejeon, Republic of Korea

**Keywords:** *Kingella kingae*, immunoinformatics, chimeric vaccine, epitope mapping, bioinformatics

## Abstract

*Kingella kingae* is a Gram-negative bacterium that primarily causes pediatric infections such as septicemia, endocarditis, and osteoarticular infections. Its virulence is attributed to the outer membrane proteins having implications in bacterial adhesion, invasion, nutrition, and host tissue damage. TonB-dependent receptors (TBDRs) play an important role in nutrition and were previously implicated as vaccine targets in other bacteria. Therefore, we targeted the conserved β-barrel TBDR domain of these proteins for designing a vaccine construct that could elicit humoral and cellular immune responses. We used bioinformatic tools to mine TBDR-containing proteins from *K. kingae* ATCC 23330 and then predict B- and T-cell epitopes from their conserved β-barrel TDR domain. A chimeric vaccine construct was designed using three antigenic epitopes, covering >98% of the world population and capable of inciting humoral and adaptive immune responses. The final construct elicited a robust immune response. Docking and dynamics simulation showed good binding affinity of the vaccine construct to various receptors of the immune system. Additionally, the vaccine was predicted to be safe and non-allergenic, making it a promising candidate for further development. In conclusion, our study demonstrates the potential of immunoinformatics approaches in designing chimeric vaccines against *K. kingae* infections. The chimeric vaccine we designed can serve as a blueprint for future experimental studies to develop an effective vaccine against this pathogen, which can serve as a potential strategy to prevent *K. kingae* infections.

## 1 Introduction

*Kingella kingae* is a Gram-negative bacterium that was first identified in 1960 (Lebel et al., 2006). It was initially considered a commensal organism of the upper respiratory tract (Al-Qwbani et al., 2016). However, subsequent studies have shown that it can cause a range of infections, particularly in children under the age of 5 years (Williams et al., 2014). Currently, it is considered an emerging pathogen in the field of pediatrics (Dubnov-Raz et al., 2010). The bacterium’s virulence is attributed to its ability to adhere to host cells, invade host tissues, and damage host cells (Porsch, 2022). Infections caused by it are usually localized and can involve various organs, such as the joints, bones, and heart (Ceroni et al., 2013). The most common manifestation of its infection is osteoarticular infection, which can present as septic arthritis or osteomyelitis (Lundy and Kehl, 1998). The infection can also affect the heart valves and cause endocarditis, which is a serious and potentially life-threatening condition (Taubert and Dajani, 2001). Prompt diagnosis and appropriate treatment are crucial to prevent complications and ensure a good clinical outcome, while prevention through vaccine is a better strategy. However, to date, no vaccine exists against this bacterium.

TonB-dependent receptors (TBDRs) are outer membrane proteins found in many Gram-negative bacteria that play a key role in the uptake of essential nutrients, such as iron and vitamin B12, from the host environment (Blanvillain et al., 2007; Moynié et al., 2017). TBDRs are highly conserved among bacteria and are essential for bacterial survival and growth, making them attractive targets for the development of vaccines (Grassmann et al., 2017; Bettin et al., 2022). One advantage of targeting TBDRs is that they are expressed on the outer surface of the bacterial cell, making them accessible to antibodies and other immune system components. Several studies have investigated the potential of TBDRs as vaccine candidates, with promising results. For example, a vaccine based on the TBDR of the pathogenic bacterium *Haemophilus influenzae* was shown to be highly effective in preventing infection in a mouse model (Webb and Cripps, 1999). Other studies have focused on the development of vaccines targeting TBDRs in pathogenic bacteria such as *Aeromonas hydrophila* (Abdelhamed et al., 2017)*, Mycobacterium bovis* (Bettin et al., 2022), *Pseudomonas fluorescens* (Hu et al., 2012), and *Acinetobacter baumannii* (Yang et al., 2017).

Traditional vaccine development approaches can be time-consuming and costly, often taking several years and requiring large amounts of resources (Mahoney and Maynard, 1999). Immunoinformatics approaches can accelerate the vaccine design process and reduce the cost by predicting epitopes that are likely to be effective (Kazi et al., 2018). The utilization of immunoinformatics allows researchers to analyze vast amounts of genomic and proteomic data to identify antigenic epitopes with desirable characteristics, such as high immunogenicity, conservation across strains, and binding affinity to immune receptors. By narrowing down the pool of potential epitopes, immunoinformatics streamlines the selection process, enabling more focused and efficient experimental validation. Chimeric vaccines, which are composed of multiple antigenic epitopes, have been widely used in infectious disease research and developed using immunoinformatics (Rahmani et al., 2019; Mishra et al., 2022). Using bioinformatics tools, researchers can predict B-cell and T-cell epitopes against the protein sequence of choice, to design a chimeric vaccine and predict the safety and immunogenicity *in silico*, reducing the cost and time required for experimental testing (Enayatkhani et al., 2021). Several studies have demonstrated the potential of immunoinformatics approaches in designing chimeric vaccines against infectious agents. For instance, Kota et al. (2021) identified B- and T-cell epitopes from the toxoid and hemolysin protein of *Staphylococcus aureus* to design a chimeric vaccine. The vaccine was predicted to be highly immunogenic and provided 83% direct and 50% passive immunization in a murine model. Another study identified MHC-binding epitopes from a Lom-like protein, a putative pilin subunit, and a section of the type III secretion structural protein EscC from the *Escherichia coli* O157:H7 (García-Angulo et al., 2014)*.* The chimeric vaccine was designed and tested in mice, as well as through other experimental assays. The vaccine induced cytokine response and reduced colonization of the pathogen in a mouse model. Similarly, immunoinformatics-based predictions for the dengue virus vaccine led to good immune response mapping in a rabbit model (Kaushik et al., 2022).

Hence, designing a chimeric construct targeting the *K. kingae* TDR domain is assumed to be a promising approach for developing a vaccine against strains of this pathogen. Therefore, the objective of this study was to design a vaccine construct against *K. kingae* using a computational approach. Prediction of antigenic epitopes that are likely to be recognized by the immune system was followed by construct design. The best immunogenic construct was docked with immune receptors and immune response simulation. This bioinformatics-aided vaccine design approach can accelerate the vaccine design process to provide protection against *K. kingae* and reduce the cost of development.

## 2 Materials and methods

### 2.1 Target sequence retrieval

**FIGURE 1:**
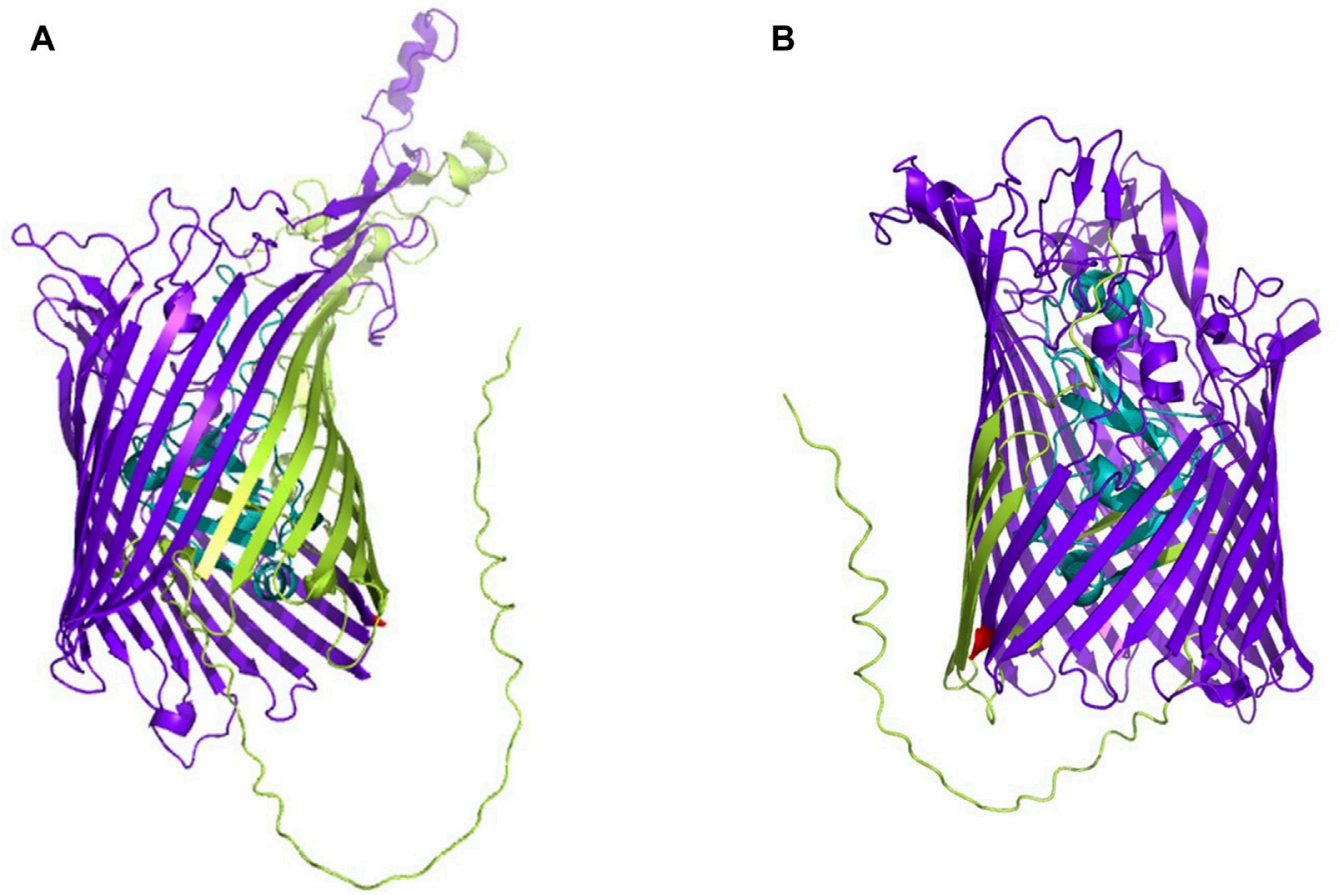
3D structures of **(A)** TbpA (https://alphafold.ebi.ac.uk/entry/F5S962; retrieved 18 March 2023) and **(B)** hpuB (https://alphafold.ebi.ac.uk/entry/F5S5I2; retrieved 18 March 2023) of *K. kingae*, with the larger TBDR β-barrel domain shown in bluish purple and the smaller TonB plug domain shown in teal. The rest of the sequence is shown in yellowish green.

The whole proteome of *K. kingae* ATCC 23330 (GenBank accession: AFHS01000057.1; comprising 3,995 sequences) was scanned against the TonB-dependent receptor domain (Pfam ID: PF00593; length = 553 amino acids) using BLAST (https://blast.ncbi.nlm.nih.gov/Blast.cgi; accessed 18 March 2023) (Altschul et al., 1997). Three protein sequences, comprising two homologs of TbpA (accession: EGK07547.1; WP_257003592.1) and an hpuB (accession: WP_003785727.1), were obtained as significant hits. Both consisted of a TonB-dependent receptor plug domain and a TonB-dependent receptor-like β-barrel domain (Figure 1). The sequence of the larger β-barrel-like domain (Pfam ID: PF00593; Figure 1) was taken and used for creating a vaccine construct against *K. kingae* ATCC 23330.

### 2.2 Epitope mapping and construct preparation

Immunoinformatics analysis was carried out using several public servers. Antigenicity of the sequence was predicted using the VaxiJen server (http://www.ddg-pharmfac.net/vaxijen/VaxiJen/VaxiJen.html; retrieved 18 March 2023) (Doytchinova and Flower, 2007). The B-cell epitopes were predicted using the two servers ABCpred (https://webs.iiitd.edu.in/raghava/abcpred; retrieved 19 March 2023) (Saha and Raghava, 2006) and BcePred (https://webs.iiitd.edu.in/raghava/bcepred/; retrieved 19 March 2023) (Saha and Raghava, 2004). ABCpred uses an artificial neural network to predict continuous B-cell epitopes based on a dataset of 700 experimental epitopes. We took a threshold of 0.5 and a window of 16 for prediction. BcePred uses data on 1,029 B-cell epitopes and takes into account parameters like hydrophobicity and flexibility. We used a default threshold ranging from −3 to 3 for these parameters. A consensus of results from both servers was taken. T-cell epitopes were predicted using IEDB-AR (https://www.iedb.org/; retrieved 20 March 2023) (Dhanda et al., 2019) and were filtered based on their antigenicity, conservancy, and accessibility to the immune system. Overlap with B-cell epitopes was studied, and the selected ones that fulfilled all criteria were arranged in a linear order to form a chimeric vaccine.

Accurately assessing and maximizing world population coverage is essential in designing vaccines that have broad efficacy and can effectively combat infectious diseases on a global scale. It helps identify the extent to which a vaccine is effective in protecting a specific pathogen across diverse populations worldwide, using HLA allele frequencies and MHC binding data. World population coverage of these epitopes was mapped using the IEDB server (http://tools.iedb.org/population/; retrieved 21 March 2023). This server maps coverage for 115 countries, covering 21 ethnicities.

A flexible linker sequence was introduced between the epitopes to ensure that each epitope maintained its conformation and function. In total, nine constructs were created and checked for antigenicity using VaxiJen and allergenicity using AllerTOP (http://www.ddg-pharmfac.net/AllerTOP/; retrieved 22 March 2023) (Dimitrov et al., 2014) and AllerCatPro (https://allercatpro.bii.a-star.edu.sg/; retrieved 22 March 2023) (Maurer-Stroh et al., 2019). Construct no. 4 (referred to as C4 hereafter), having the best antigenicity score and a non-allergen, was taken for structure modeling.

### 2.3 Structure modeling and dynamics simulation

The 3D structure was modeled using AlphaFold v2.3.1 (Jumper et al., 2021). AlphaFold is a deep learning algorithm developed by DeepMind that predicts the 3D structure of a protein from its amino acid sequence. The final predicted structure was selected based on the conformation having the lowest energy. A per-residue estimate of its confidence on a scale from 0 to 100 was measured using a scale called pLDDT, with a value >90 referring to high accuracy, and 70 or above expected to be modeled well (Zifruddin et al., 2023). The structure was minimized using the OPLS3e force field in the software package Maestro (van Zundert et al., 2021). Bond orders were assigned using the CCD database, and Het states were generated using Epik at pH 7.0+/-2.0. Hydrogen bond assignment was carried out using PROPKA at pH 7. Heavy metals were converged to 0.3 Å. This minimization process enhances quality by removing any small stereochemical violations present and generally results in minimal differences between the predicted and relaxed structures. Molecular dynamics simulation was also conducted for 100 ns in Desmond, Schrodinger Inc. (United States). The construct was prepared by adding hydrogens, assigning bond orders, and adding disulfide bonds. Refinement was carried out at pH 7 in the PROPKA module. Restrained minimization was carried out by converging heavy atoms to 0.30 Å. The solvation model was TIP3P, box=orthorhombic, box size calculation method: Buffer and neutralization was carried out by adding counter ions (Na+ or Cl-according to requirement). Simulation parameters were force field: OPLS2005 force field; Ensemble: NVT; system temperature: 300 K; pressure: 1.01 (bar); recording interval: 100 ps. The rest of the options were default. RMSD and RMSF were obtained along with the secondary structure over a time course of 100 ns.

### 2.4 Molecular docking

The binding affinity of the chimeric vaccine to the immune receptors (HLA alleles and TLRs), specifically known to interact with the adjuvant used in C4, was carried out using ClusPro (Kozakov et al., 2017). TLRs are involved in recognizing pathogens, while HLAs help present antigens to T cells. Among TLRs, TLR-1, TLR-1/2, and TLR-4 were included, while HLA receptors included HLA-A*0201, HLA-B*5301, HLA-CW3, HLA-DRA1, HLA-DRB1, HLA-DP1, HLA-DP2, HLA-DQA1, and HLA-DQB1. ClusPro is based on a fast Fourier transform (FFT) algorithm that uses rigid-body docking to generate a large number of candidate models for protein–protein complexes. It then uses a clustering algorithm to group these candidate models into clusters based on their similarity, and it ranks the top models by their calculated binding energy (Comeau et al., 2004). Interaction prediction could help infer binding between C4 and human immune receptors, and the subsequent initiation of immune response. Post-docking interactions were visualized using Protein Interactions Calculator (PIC) (http://pic.mbu.iisc.ernet.in/; accessed 22 March 2023) (Tina et al., 2007).

Normal mode analysis is a computational method used to study the collective vibrational motions of a protein or other biomolecules (Case, 1994). It can provide insights into protein dynamics and conformational changes. WEBnm@ (http://apps.cbu.uib.no/webnma3/; accessed 23 March 2023) (Tiwari et al., 2014) was used to study conformational changes and for comparing unbound and bound C4 via the lowest-energy collective vibrational modes, such as global twisting or bending motions. Atomic displacement graphs in several modes and a correlation matrix were used to observe the changes in bound and unbound C4. MD simulation was carried out for all the receptors bound with C4. Parameters were the same as described in Section 2.3, except for the time duration being reduced to 50 ns due to the complexity of the molecular system.

### 2.5 Immune response simulation

C-IMMSIM (Castiglione and Bernaschi, 2004) was used to simulate the interactions between the vaccine construct, immune cells, antigens, and cytokines, and to study the dynamics of the immune response under different conditions. It is a C++-based agent-based modeling framework that integrates both cellular and molecular components of the immune system. Hence, the response of various immune cells, such as T cells, B cells, and dendritic cells, to C4 and *K. kingae* TbpA and hpuB challenge was attempted. For most of the vaccines currently in use, 4 weeks is the minimum recommended time between the first and second doses (Saha et al., 2021). We utilized the same values. The entire simulation ran for 1,000 time steps, which are ∼11 months (1 timestep=8 h). Two vaccine injections were given on days 1 and 30 (i.e., after 1 month), followed by the bacterial challenge on day 240 (i.e., after 8 months since vaccine initiation and 7 months after the end of vaccination) to check the efficacy of the vaccination process. Host HLA selection was A0101, A0102, B0702, B0704, DRB101, and DRB102. The rest of the parameters were as described previously (Rahman et al., 2020).

### 2.6 Cloning

*In silico* cloning is a computational method used to predict the optimal restriction enzyme sites and sequence overlap for the insertion of a DNA sequence of interest into a cloning vector (Celie et al., 2016). It is carried out to design the cloning strategy and is helpful in guiding wet laboratory experiments. JCat (Grote et al., 2005) was used for optimization of the codon usage of C4 to enhance its expression in *E. coli.* Codon optimization was attempted because it can lead to higher yields of the antigen protein and a stronger immune response in the host (Ramakrishna et al., 2004). The C4 protein sequence was first reverse-translated to the DNA sequence, and then, the codon usage was adjusted to match the preferred codon usage of *E. coli.* A suitable pET vector was then selected for cloning this sequence in SnapGene software (available at https://www.snapgene.com/) based on optimal restriction enzyme sites for C4. It was then cloned, and the image was saved in .jpeg format.

## 3 Results

### 3.1 Construct assembly

In total, 538 16-mer B-cell epitopes were obtained from the ABCpred server, and 11 epitopes were obtained from the BcePred server. Additionally, 29,403 epitopes were predicted for MHC-I and 14,553 for MHC-II. Finalization was carried out based on various properties like immunogenicity and sequence coverage. The final MHC-I and MHC-II epitopes were checked for cumulative population coverage. This was carried out to identify epitopes that are likely to be recognized by a large proportion of the population to design a vaccine effective in a broad range of individuals. By targeting epitopes that are highly conserved and commonly presented by MHC molecules, we can increase the likelihood of generating a robust and broadly protective immune response. According to the IEDB server, the cumulative MHC-I and MHC-II epitope population coverage of the world was predicted to be 98.55% (Supplementary Figure S1). This means that more than 98% of the world’s population is likely to have at least one MHC-I or MHC-II allele that can bind to this set of epitopes. Achieving high world population coverage is a desirable goal in vaccine design as it aims to ensure that a significant majority of individuals, regardless of geographic location, genetic variations, or other demographic factors, can mount an effective immune response against the targeted pathogen. A vaccine with high world population coverage shows potential to reduce the overall burden of the disease, prevent transmission, and contribute to global public health.

Finally, three overlapping validated epitopes (Table 1) were selected for designing the vaccine constructs (Supplementary Table S1). The overlap with B-cell epitopes was then studied to select those that fulfilled all criteria and arranged in a linear order to form a chimeric vaccine. To ensure the stability and functionality of the epitopes, a flexible linker sequence was introduced between them, along with adjuvants like β-defensin and flagellin. Adjuvants are added to enhance the immunogenicity of the vaccine and stimulate the immune response (Bastola et al., 2017). Nine different vaccine constructs were prepared and checked for antigenicity and allergenicity using VaxiJen, AllerTOP, and AllerCatPro servers. C4, which had the best antigenicity score and was non-allergenic, was selected for structure modeling.

**TABLE 1:** Final epitopes used for preparing the vaccine construct.

Serial no.	Sequence	Length (aa)	Conservancy (%)	Toxicity
1	DQCNYRGNSENYSDCSGRVIKGS	23	100	Non-toxic
2	LEASYFNNDYRDLITFGCQI	20	100	Non-toxic
3	NARLGGVNVLGKIYWNG	17	100	Non-toxic

### 3.2 Structure modeling and simulation

**FIGURE 2:**
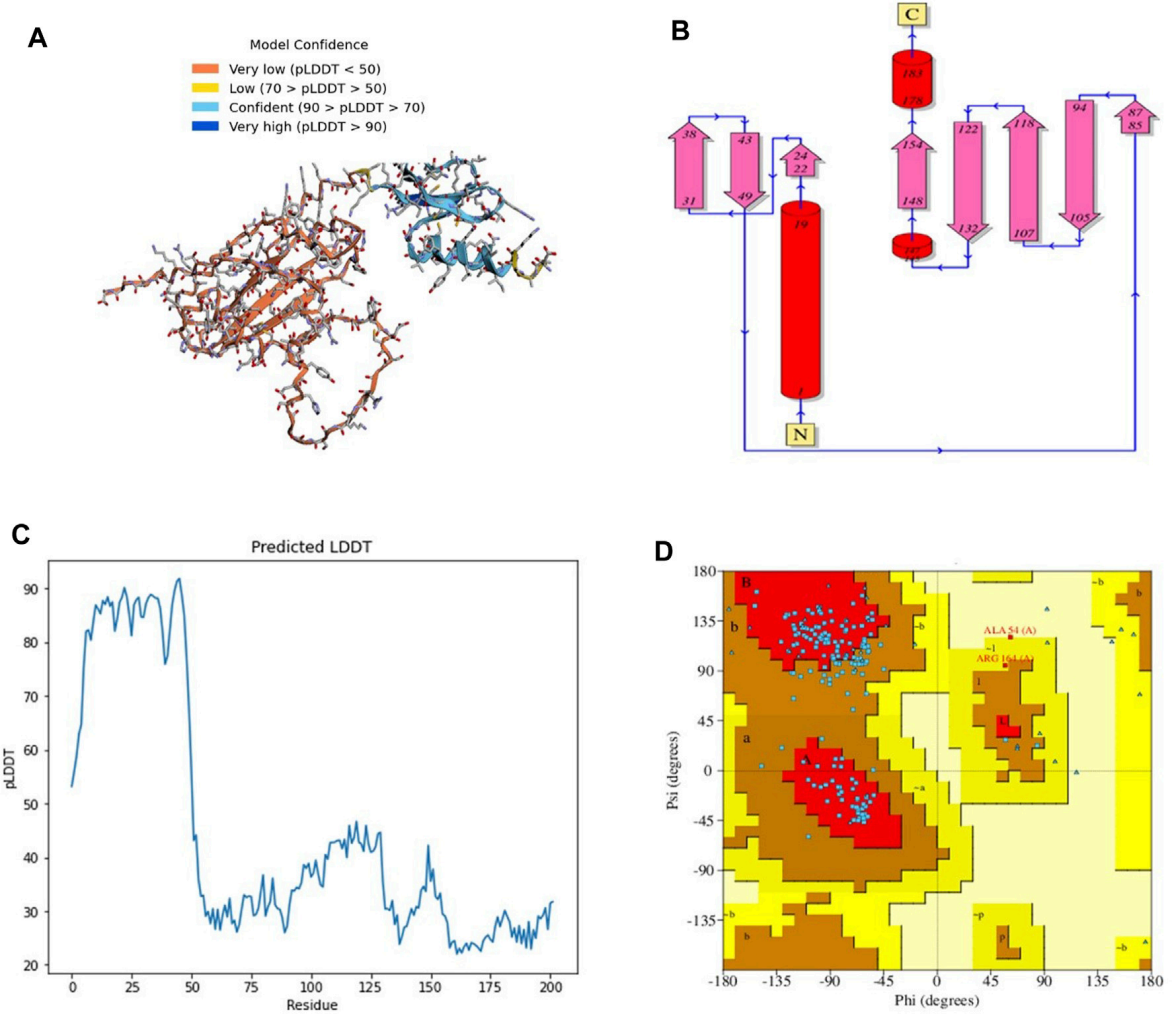
**(A)** 3D model of C4 colored according to model confidence. **(B)** 2D depiction of C4 topology. **(C)** LDDT plot of the predicted structure. **(D)**. Ramachandran plot of the predicted structure.

The 3D structure of a vaccine construct is important because it can provide insights into its function, interactions with other molecules, and potential immunogenicity. The 3D structure (Figure 2A) comprised 2 sheets, 5 β-hairpins, 3 β-bulges, 8 strands (Figure 2B), 3 helices, 14 β-turns, 15 gamma turns, and 4 disulfides. Disulfides are covalent bonds between two cysteine residues that help stabilize the protein structure and prevent degradation (Trivedi et al., 2009).

The first 50 residues modeled had a high confidence level (Figure 2C), while the overall Ramachandran plot (Figure 2D) showed 74% residues in highly favored regions, 24% in allowed regions, and the rest in disallowed regions.

**FIGURE 3:**
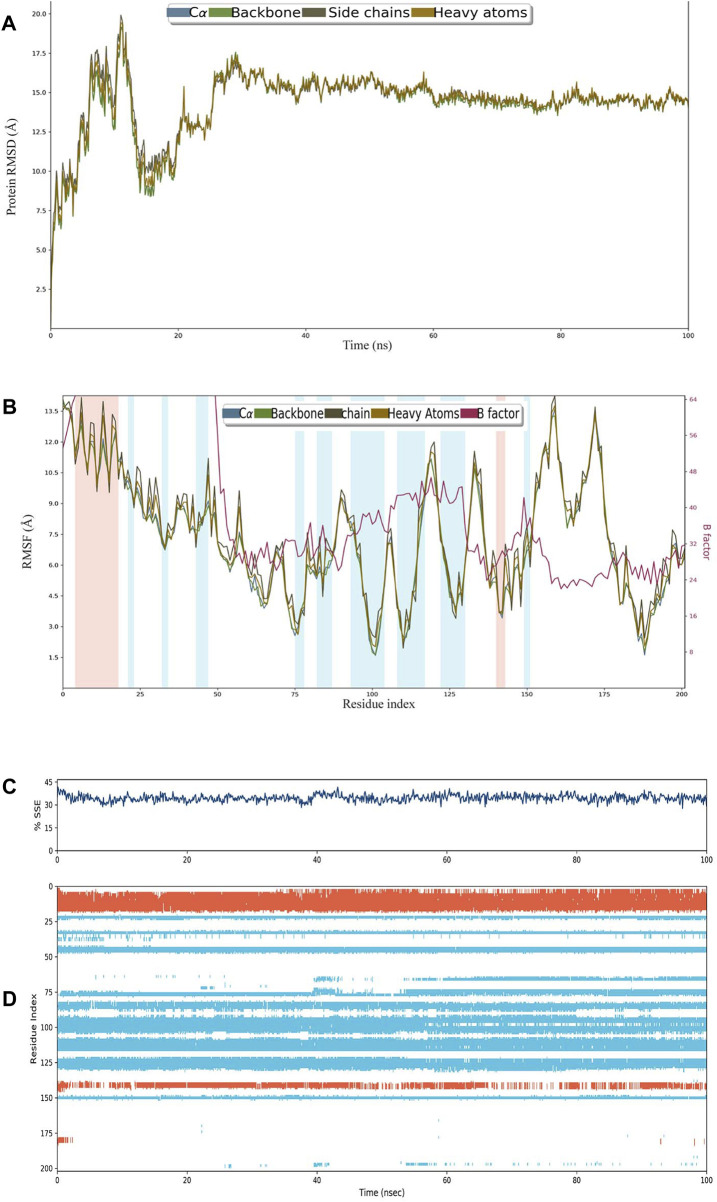
**(A)** RMSD plot of construct C4. **(B)** RMSF plot of C4. **(C)** Secondary structural elements (SSEs) like α-helices and β-strands throughout the simulation. **(D)** Residue scale SSE (α-helices shown in orange; β-strands shown in sky blue) assignment over 100 ns.

The construct stabilized after 30 ns of simulation, and its RMSD remained approximately 15 Å after 60 ns (Figure 3A). RMSF remained less than 12 Å for most of the residues, and there was very little variation between heavy atoms, side chains, and the backbone (Figure 3B). The percentage of helices was 8.89 and that of strands was 25.58%, making SSE>30% of the residues over the course of simulation (Figure 3C). A major portion of the structure retained their composition of helices and sheets throughout the simulation (Figure 3D).

### 3.3 Docking and dynamics simulation analysis

ClusPro is a widely used docking program that predicts the binding affinity and orientation of two protein structures (Kozakov et al., 2017). Here, it was used to predict the interaction between the vaccine construct and the target proteins TLR1, TLR2, and TLR4, and various HLA molecules. The results showed that C4 has a high predicted binding affinity with all the target proteins, and docking scores range from −1550.0 to −1017.4, indicating strong interactions (Table 2). The lowest docking score was obtained with the protein TLR2, followed by TLR1 and TLR4, which suggests that C4 may have a high binding affinity for these receptors. The HLA molecules tested also showed a good interaction with C4, with docking scores ranging from −1709.4 to −1162.9, and binding is likely to potentially increase C4 efficacy as a vaccine. Previously, the interaction of TLR4 with the vaccine construct designed against *Haemophilus influenzae* (ClusPro score: −1035.2 kcal/mol) and *Streptococcus pneumoniae* (ClusPro score: −1150.5 kcal/mol) was reported to be robust (Mazumder et al., 2023). Our score was higher than the score in these studies, indicating a highly stable binding affinity (AlChalabi et al., 2022). Similarly, the binding interaction score of the vaccine construct designed against *Candida tropicalis* and HLA-A*0201 has been reported as − 1178.4 kcal/mol (Akhtar et al., 2022), whereas it was −1218.9 kcal/mol in this study, indicating good binding affinity. It is important to ensure the reliability and accuracy of computational methods in determining binding affinities. For this purpose, we incorporated cross-validation of docking calculations by introducing a control (TLR1–TLR2 interaction; PDB ID: 2Z7X) with a strong experimentally known binding affinity (Jin et al., 2007) and compared against the docking prediction for our construct. The TLR1–TLR2 interaction cluster with the largest number of members had the lowest energy of −936.5 kcal/mol. Energy values of C4 binding with immune receptors were much lower than this value, indicating more stable and possibly robust interactions. The stability of the interactions between C4 and immune receptors is an important factor in determining the effectiveness of the immune response. Lower energy values imply stronger binding between C4 and the receptors, suggesting a higher likelihood of successful recognition and activation of the immune system. Robust interactions can trigger a more vigorous immune response, which is desirable in combating pathogens.

**TABLE 2:** HLA and TLR interaction statistics with the designed vaccine construct. 3D depiction of interactions is shown in Supplementary Figure S3.

Serial no.	PDB ID	Chain	Name	Lowest docking score with C4	Hydrophobic interactions	No. of hydrogen bonds in main chains	Ionic bonds	Aromatic interactions
1	6NIH	A	TLR1	−1102.7	14	1	8	4
2	2Z80	A	TLR1/2	−1017.4	4	0	5	1
3	3FXI	A	TLR4	−1355.7	9	9	10	0
4	1AKJ	A	HLA-A*0201	−1218.9	12	4	11	2
5	1A1M	A	HLA-B*5301	−1162.9	9	0	7	1
6	1EFX	A	HLA-CW3	−1261.0	8	0	9	0
7	1A6A	A	HLA-DRA1	−1399.8	19	0	6	2
8	1A6A	B	HLA-DRB1	−1489.0	32	6	3	10
9	3LQZ	A	HLA-DP1	−1494.4	20	2	8	6
10	3LQZ	B	HLA-DP2	−1550.0	23	1	4	6
11	1JK8	A	HLA-DQA1	−1709.4	29	3	9	4
12	1JK8	B	HLA-DQB1	−1408.4	13	5	5	2

Delving into the details of interactions, C4 formed a significant number of hydrophobic interactions and hydrogen bonds with all interactors, which are crucial for stabilizing the interaction. A smaller number of ionic bonds and aromatic interactions were also observed, which contributed to the stability of the interaction. The highest number of hydrophobic interactions were observed for HLA-DRB1 (*n*= 32), the largest number of hydrogen bonds was observed for HLA-DP2 (*n* = 23), and maximum ionic interactions were detected for HLA-DRB1 (*n* = 3). C4 also formed aromatic interactions with some interactors, with the highest number observed for HLA-DRB1 (*n* = 10). Aromatic interactions are relatively weaker than hydrophobic interactions and hydrogen bonds, but they can add to the strength of the interaction. Normal mode analysis for C4 showed major fluctuations around residues 30 and 40, and between residues 160 and 170 (Supplementary Figure S2), while binding appeared restrained for the TLR4 receptor. Displacements were also observed for HLA-DRA1 and HLA-DQB1.

**FIGURE 4:**
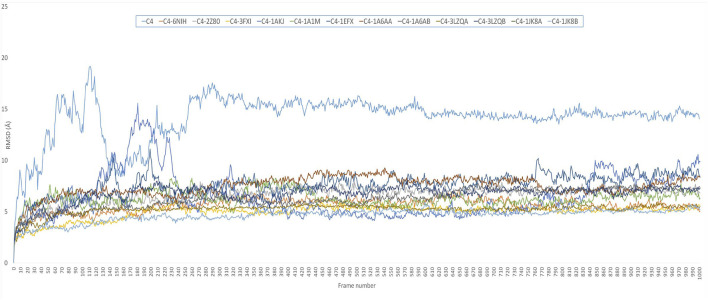
RMSD plot of 50-ns simulation showing C4=vaccine construct; C4-6NIH=vaccine–TLR1 complex; C4-2Z80=vaccine–TLR1/2 complex; C4-3FXI=vaccine–TLR4 complex; C4-1AKJ=vaccine–HLA-A*0201 complex; C4-1A1M=vaccine–HLA-B*5301 complex; C4-1EFX=vaccine–HLA-CW3 complex; C4-1A6AA=vaccine–HLA-DRA1 complex; C4-1A6AB=vaccine–HLA-DRB1 complex; C4-3LZQA=vaccine–HLA-DP1 complex; C4-3LZQB=vaccine–HLA-DP2 complex; C4-1JK8A=vaccine–HLA-DQA1 complex; and C4-1JK8B=vaccine–HLA-DQB1 complex.

Comparative MD simulation (Figure 4) of the C4 vaccine construct and bound immune receptors revealed that the RMSD of the complexes (Supplementary Figure S4) was much lower than that of the vaccine construct alone. This suggests that binding to the immune receptors stabilizes the structure of the vaccine construct. RMSD values varied among different immune receptors. HLA-DP1, HLA-DQA1, and TLR-4 had comparatively low average RMSD values (less than 6), indicating that the complexed vaccine remained relatively stable when bound to these receptors, while TLR1/2, HLA-B*5301, HLA-DRA1, HLA-DRB1, HLA-DP2, and HLA-CW3 complexed with the vaccine had higher average RMSD values (less than 8), suggesting that the complex with these receptors experienced more structural fluctuations. HLA-A*0201 and HLA-DQB1 exhibited a more dynamic behavior than the rest of the immune receptors. RMSD of HLA-A*0201-C4 reached up to 15 Å at approximately 10 ns and then stabilized for some time for less than 8 Å until 40 ns. It again increased to more than 8, and the trend continued upward. HLA-DQB1 reached up to 12 Å after 20 ns and then stabilized but remained higher than 10.5 Å, peaking toward 12 Å after 35 ns. The initial increase in RMSD indicates structural adjustments or fluctuations as they accommodate the vaccine construct. The subsequent stabilization suggests a period of relative structural stability, but this stability is at a higher RMSD level, indicating that these receptors undergo some level of structural change even in their stable states and as time progresses. The results are in alliance with the findings of normal mode analysis, where HLA-DQB1 showed higher displacement.

**FIGURE 5:**
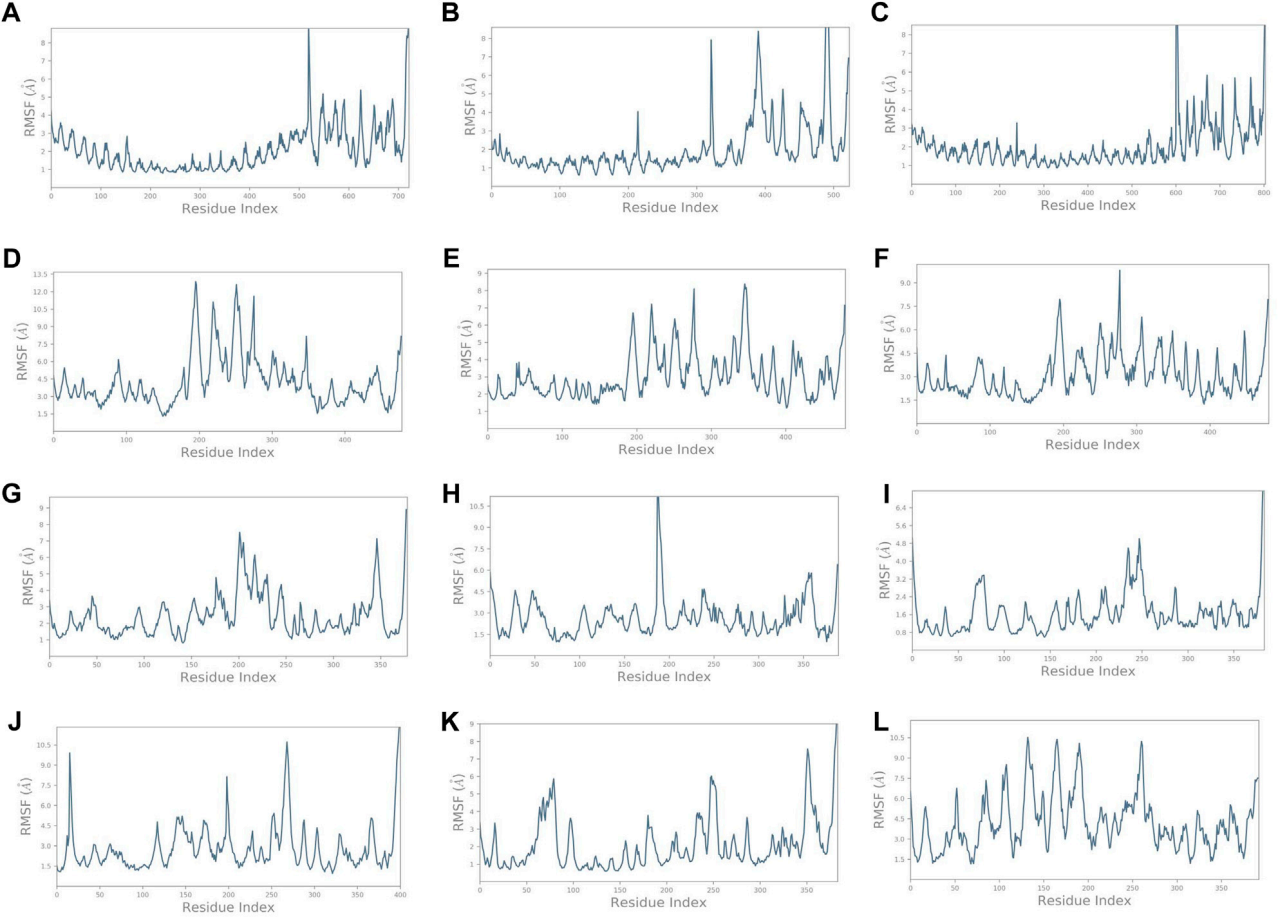
RMSF plots of the vaccine construct bound with **(A)** TLR1. **(B)** TLR1/2. **(C)** TLR4. **(D)** HLA-A*0201. **(E)** HLA-B*5301. **(F)** HLA-CW3. **(G)** HLA-DRA1. **(H)** HLA-DRB1. **(I)** HLA-DP1. **(J)** HLA-DP2. **(K)** HLA-DQA1. **(L)** HLA-DQB1.

Similarly, it was noted that the RMSF varied for complexes (Figure 5) compared to the vaccine construct alone (Figure 3B). Findings provide insights into the dynamic nature of the interaction between these immune receptors and the vaccine construct, suggesting that the receptors undergo structural adaptations during the simulation. The initial residue portion of the graph shows the immune receptor RMSF, while the last 200 residues depict the vaccine construct C4. The RMSF of TLR1 was less than 3 Å while that of the construct C4 was less than 5 Å on average. It exceeded 4.5 Å on average in the unbound form. It was even lower for TLR1/2, TLR4, HLA-DRA1, HLA-DRB1, and HLA-DP1. HLA-B*5301, HLA-CW3, and HLA-DQB1 showed higher fluctuations. These observations highlight the dynamic nature of the interaction between the immune receptors and the vaccine construct. Different receptors exhibit varying levels of stability and flexibility during the simulation, which suggests that the dynamic nature of receptor–vaccine interactions observed in the simulation may have implications for the effectiveness of a vaccine. A stable interaction with immune receptors could be important for eliciting a strong and targeted immune response, which was depicted by the receptors TLR1/2, TLR4, HLA-DRA1, HLA-DRB1, and HLA-DP1.

### 3.4 Immune response simulation

**FIGURE 6:**
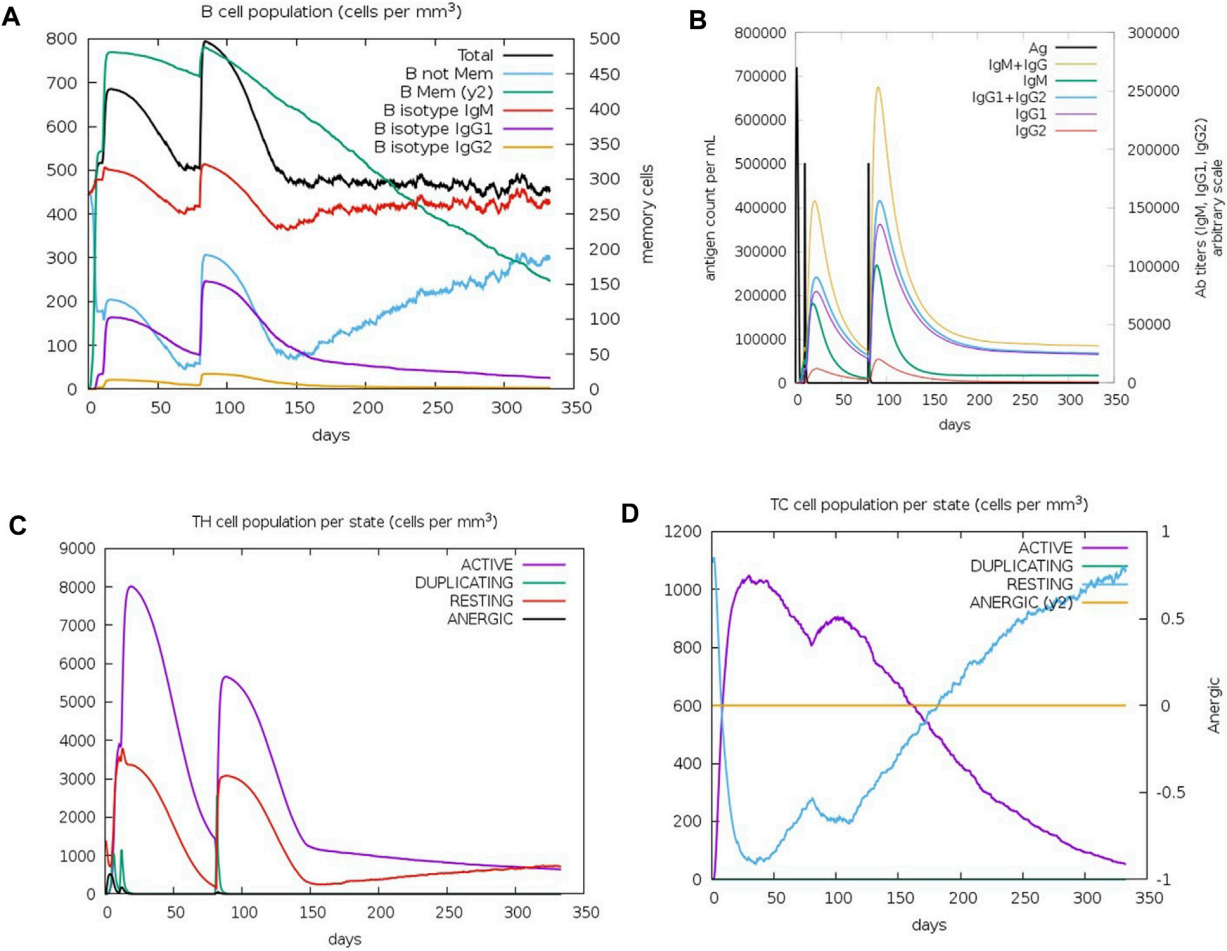
Immune cell population post-C4 injection. **(A)** Graph depicting the change in the B-cell population. **(B)** Varied antibody titer. **(C)** Altered TH cell population. **(D)** TC cell population for active, resting, duplicating, and anergic states.

The C-IMMSIM results describe the efficacy of the vaccination process against *K. kingae* infection, attempted by injecting tbpA and hpuB proteins after 7 months of vaccination. Memory B cells kept decreasing after 3 months, while non-memory B cells kept increasing after 5 months (Figure 6A). IgM (first antibody to be produced in response to an initial exposure to a pathogen) and the total B-cell population plateaued (at ∼500 cells per mm^3^) 4 months after vaccination. IgM is the first antibody that is produced in response to an initial exposure to a pathogen, and the plateauing of its levels and that of the total B-cell population suggests that the immune system mounted a robust initial response to the vaccine, but this response might not continue to increase or remain at a high level over an extended period.

The immunoglobulin population stabilized after 4 months, with the antibody titer count of IgG1+ IgG2 (antibodies that are involved in long-term immunity) and IgM+IgG being approximately 20,000 for 100,000 antigens per ml (Figure 6B), indicating that the immune system has produced a significant amount of antibodies against the pathogen, which should provide strong protection. T-helper (TH) cells reached up to 8,000 cells per mm^3^ after 20 days of the first injection (Figure 6C), with a doubling of TH1-active cells. Resting and active TH cell population stabilized at 1,000 cells per mm^3^ after 300 days. TH cells have a relatively short lifespan and are continuously produced and replaced by the immune system. Therefore, the stabilization of the TH cell population may not necessarily imply a lack of long-term protection. Immune response to the vaccine was not uniform across all T-cell populations. The TC memory cell population remained stable, while non-memory cells fluctuated, with active cells decreasing and resting cells increasing after 4 months of vaccination (Figure 6D).

### 3.5 Cloning of C4

**FIGURE 7:**
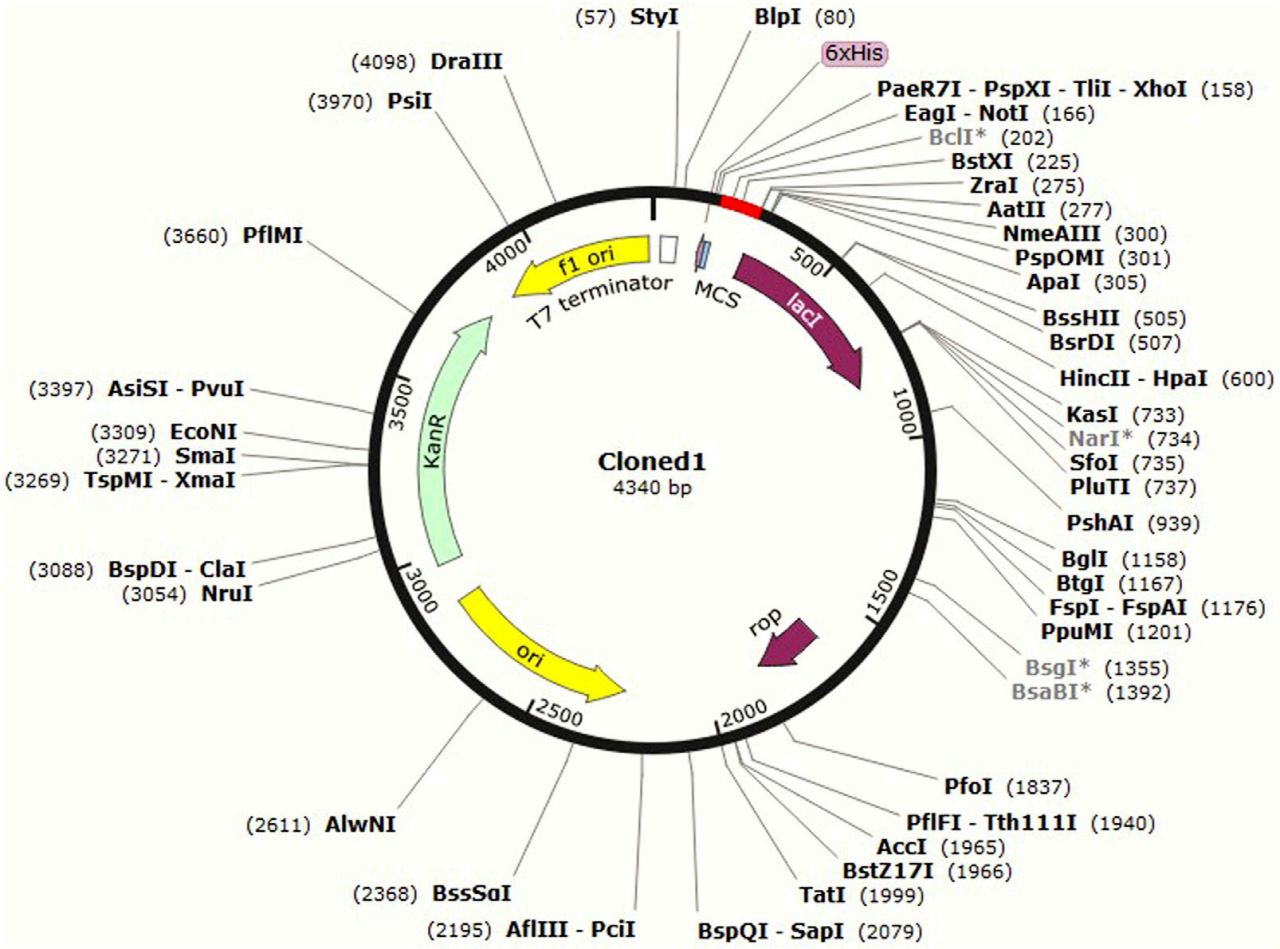
Cloned C4 in the pET-30a+ vector, shown in red.

*In silico* cloning allows researchers to test different cloning strategies and find the best combination of restriction enzymes and vector sequences to use without the need to perform multiple trial-and-error experiments in the laboratory. This can save time and resources in the cloning process. C4 was cloned in the pET-30a (+) vector in *E. coli* K12 (Figure 7).

The pET-30a (+) vector is commonly used in molecular biology as it contains several useful features for protein expression in *E. coli*, such as strong promoters, a His-tag for protein purification, and antibiotic resistance markers for selection. The choice of HindIII and BstEII enzymes was based on their ability to generate compatible overhangs or “sticky ends” that can be easily ligated to the corresponding ends of the pET-30a (+) vector. C4 was then inserted in the vector and cloned in *E. coli.* This approach of *in silico* cloning could help save time and money by allowing the design and test of virtual constructs before actually synthesizing or cloning them in the laboratory.

## 4 Discussion

Proteins known to be involved in iron acquisition in bacteria are potential targets for vaccine development (Schryvers and Stojiljkovic, 1999; Glanfield et al., 2007; Ismail et al., 2022). We aimed to utilize the conserved domain of TBDRs involved in iron and nutrient acquisition for vaccine design against the *K. kingae* reference strain ATCC 23330. For this purpose, we scanned the proteome of *K. kingae* and identified two homologs of the TBDR protein (TbpA and hpuB) comprising the TBDR β-barrel domain. Previous studies identified TbpA and hpuB proteins as important virulence factors in other bacteria, such as *Actinobacillus pleuropneumoniae* (Klitgaard et al., 2010) and *Neisseria gonorrhoeae* (Lin et al., 2022). Finney et al. showed that antibodies against these proteins can protect against *Neisseria lactamica* meningococcal infection in rabbits (Finney et al., 2008). The TBDR transporter of *Neisseria gonorrhoeae,* i.e., TbpB, has also been targeted for vaccine design, in addition to TbpA, and shown to invoke an immune response in mice (Greenawalt, 2021). Additionally, several vaccine candidates targeting TBDRs have been developed for various bacterial pathogens, including *Acinetobacter baumannii*, *Pseudomonas aeruginosa*, and *Klebsiella pneumonia* (Wang et al., 2021). Therefore, we used the beta-barrel domain of TBDR from the *K. kingae* reference strain ATCC 23330 for creating a vaccine construct by predicting B- and T-cell epitopes. The selected ones fulfilling all criteria were manually arranged with adjuvants, linkers, etc., to assemble a vaccine construct. The generation and use of B- and T-cell epitopes through computational servers is an established strategy for fabricating a vaccine construct capable of inducing protective immune responses against a pathogen (Wang et al., 2020). B-cell epitopes are regions on the surface of a pathogen that can be recognized by antibodies (Ponomarenko and Van Regenmortel, 2009), while T-cell epitopes are fragments of a pathogen’s proteins that can be recognized by T cells (Khatoon et al., 2017). By including both types of epitopes in a vaccine construct, the immune system is activated to generate a robust and specific immune response. Population coverage analysis (>98%) of the prioritized epitopes suggests that the antigens are likely to be effective against a majority of individuals within the world population.

Adjuvants enhance the immune response (Bastola et al., 2017), and the best construct in this study (C4), with the highest antigenicity, had a β-defensin adjuvant. β-defensin is an antimicrobial peptide that can stimulate the innate immune system (dendritic cells and macrophages) and enhance antigen-specific immune responses (Shelley et al., 2020). β-defensin may be induced by inflammatory mediators in the body and has broad-spectrum antimicrobial activity against bacteria, viruses, and fungi (Gao et al., 2021). After assembling the construct, it was subjected to 3D structure modeling to analyze its capability to bind the immune system components like HLA alleles and TLRs, and invoke an immune response. Recent advances in deep learning techniques have led to the development of AlphaFold (Jumper et al., 2021), an AI-based tool for predicting protein structures with remarkable accuracy. We attempted 3D structure modeling of C4 using AlphaFold, and it was noted that the structure consisted of four natural disulfide bonds. Disulfide bonds are formed between two cysteine residues, where the thiol groups (-SH) on each cysteine react to form a covalent bond (-S-S-) under oxidizing conditions (Saito et al., 2003; Barford, 2004). These are crucial for maintaining the stability and conformation of the protein in the context of vaccine design and can be engineered into the construct to enhance its stability and immunogenicity (Scheiblhofer et al., 2017). However, in C4, these were present naturally, depicting a stable construct. C4 was then docked with critical components of the immune system using ClusPro (Comeau et al., 2004). ClusPro is a widely used tool for predicting protein–protein interactions, and it has been applied in several studies to predict the interaction between vaccine constructs and immune system components. One such example is a study between TLR9, HLA class I/II alleles, and a vaccine construct against Kaposi’s sarcoma (Chauhan et al., 2019), while another study on *Candida auris* vaccine design reported docking between TLR5 and MHC class-II HLA DRB_0101 with scores in a similar range (Akhtar et al., 2021). The docked results indicated that C4 has a high binding affinity with all the target proteins, with hydrophobic interactions and hydrogen bonds stabilizing the interactions. However, it is important to consider that binding affinity alone does not guarantee an effective immune response or protection against a specific pathogen. Other factors, such as the overall immunogenicity of the antigen, the activation of appropriate immune pathways, and the generation of memory responses, are also critical in achieving a robust and protective immune reaction.

Normal mode analysis was then performed to investigate the flexibility and dynamics of C4 with immune components. Low-frequency vibrations, representing the collective movements of the atoms in the protein, were calculated, and the results showed that C4 had major fluctuations around residues Pro30 and Thr40, and between the residues at position 160–170 (DCSGRVIKGSG). These residues were in the loop regions and, therefore, more flexible than the rest of the structure (Supplementary Figure S5). Loop regions are segments of the protein structure that connect secondary structural elements such as ɑ-helices and β-sheets (Chavan, 2014). These regions are often more flexible and exhibit more conformational changes than the other parts of the protein (Papaleo et al., 2016). Loop regions have immunogenic characteristics and are used in vaccine design for HIV (Montero et al., 2008), *Leptotricha buccalis* (Alshammari et al., 2022), and dengue virus (Urakami et al., 2017). Previously, Ismail et al. described fluctuations in the loop regions of the vaccine construct against Enterobacteriaceae, with loops inferred to adopt a stable conformation with increasing time, during the simulation (Ismail et al., 2020). They suggested that this might help in flexible binding with host immune components. Previously, the CDR H3 loop-dominated recognition of CD4 epitopes was also reported for HIV (Pancera et al., 2017). Thus, it is inferred that these regions may become displaced and help in flexible binding with HLAs and TLRs of the immune system. The varying levels of structural stability and flexibility observed through the RMSD and RMSF plot of different receptors via MD simulation also have implications for understanding immune responses and guiding the development of vaccines with improved efficacy. The stability and specificity of these interactions may be optimized to enhance the C4-mediated immune response.

Immune response simulation analysis revealed that the vaccine construct induced a robust initial immune response against *K. kingae* infection through the introduction of tbpA and hpuB proteins in the body. The production of immunoglobulin antibodies and their subsequent stabilization, along with the doubling of TH1-active cells, indicates strong protection against the pathogen. However, the immune response was not uniform across all T-cell populations. Although the long-term protection provided by the TC memory cells remains stable, the short-term immune response decreased after a few months of vaccination. Fluctuations in some cells, e.g., the effector T cells, may indicate a natural reaction of the immune system to the vaccine and not a lack of efficacy. The immune system is complex and dynamic, and its response to a vaccine can vary among individuals and over time (Shen-Orr and Furman, 2013; Laserson et al., 2014; Brodin and Davis, 2017). Therefore, it is important to consider multiple parameters of the immune response when assessing the efficacy of a vaccine. *In silico* immune simulations can provide valuable insights into immune responses, but they cannot fully capture the complexity of the immune response (Russo et al., 2020) and, therefore, must still be validated through *in vitro* and *in vivo* experiments. Hence, further studies are needed to fully understand the dynamics of the immune response over an extended period of time for this vaccine construct against *K. kingae*.

There were several limitations to our work. *In silico* protein structure prediction is challenging, and inaccuracies in the 3D structure of the vaccine construct might have affected the reliability of docked interaction findings. Mutations in the proteins used for designing vaccines over time may alter their antigenic properties, hence compromising the reliability of our findings over time. Apart from this, biological systems are highly complex, and many factors contribute to the immune response. Vaccine efficacy depends on multiple factors, including innate and adaptive immune responses, cellular and humoral immunity, and long-term memory. Integrating all these factors accurately is difficult, and computational models may oversimplify certain reactions, leading to inaccuracies in predicting actual immune responses. Our understanding of how the immune system recognizes and responds to the computationally predicted epitopes based on molecular docking analysis might not always be accurate, and information about the induction of the desired immune response is uncertain. Despite these limitations, computational vaccine design remains a valuable tool that can significantly accelerate the vaccine development process and guide experimental research.

## 5 Conclusion

The *K. kingae* species hosts TBDRs, which acquire and transport important substances like iron and vitamins. These can be used for the design of a chimeric vaccine construct against the conserved region (existing as a motif or domain). Chimeric vaccines can reduce the risk of adverse reactions as they typically contain only selected antigenic epitopes rather than whole pathogens or toxins. Therefore, a multi-epitope vaccine construct was designed and tested for immune response elicitation through docking with immune system components and simulation. The use of computational modeling and docking studies can provide valuable insights into the interactions between vaccine components and immune system components, which can inform the design and development of more effective vaccines. RMSD and RMSF plots after MD simulation revealed that different receptors exhibited varying levels of stability and flexibility during the simulation, which depicted that C4 adapts and interacts differently with different receptors. Immune response simulation through agent-based modeling showed good binding affinity along with a robust immune response elicitation for C4 against *K. kingae* infection. However, the decrease in memory B cells may indicate that the immunity provided by the vaccine could wane over time, potentially leading to a higher risk of reinfection and the need for booster doses. Additional experimental validation through *in vitro* and *in vivo* studies is suggested to confirm the efficacy of the vaccine construct in inducing an immune response and improving the longevity of immunity.

## Data Availability

The proteome data used in this study is present in the NCBI database (GenBank accession number: AFHS01000057.1). Protein sequences for vaccine design exist in NCBI as TbpA (Accession: EGK07547.1; WP_257003592.1) and an hpuB (Accession: WP_003785727.1).
