# Supplementary material for: Chimeric vaccine design against the conserved TonB-dependent receptor-like β-barrel domain from the outer membrane tbpA and hpuB proteins of Kingella kingae ATCC 23330
Source: Front Mol Biosci. 2023 Nov 20;10:1258834. doi: 10.3389/fmolb.2023.1258834 (PMC10694214; doi:10.3389/fmolb.2023.1258834)
Supplement: Supplementary file 1 [file Table1.DOCX]

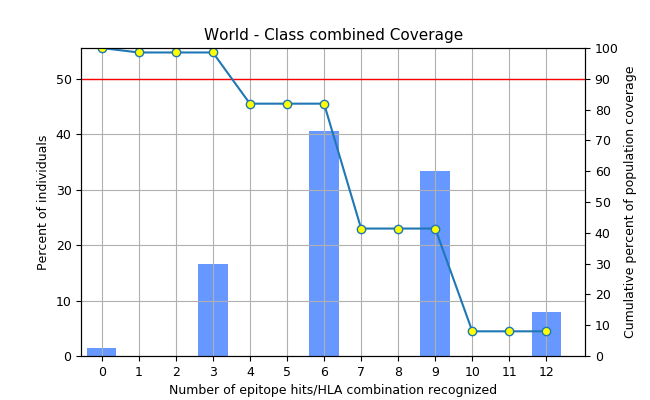


Supplementary Fig. 1. Population coverage of MHC-I and MHC-II alleles.


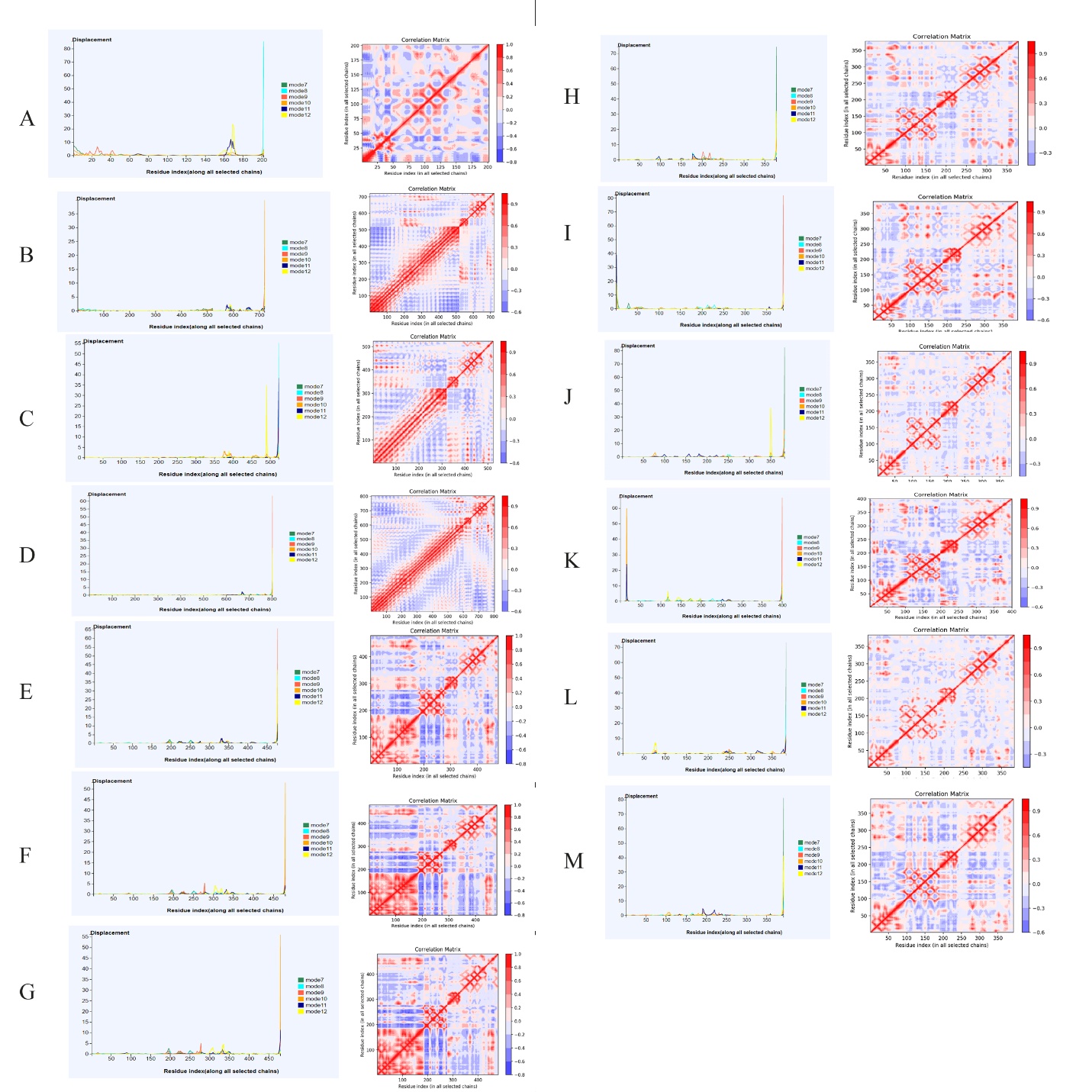


Supplementary Fig. 2. The square of the displacement of each C-alpha atom (for modes 7 to 12) with the highest values correspond to the most displaced regions. Clusters of peaks on the plots identify significantly displaced regions, while isolated peaks may reflect local flexibility in a low-density region such as the N- or C-terminus region in a structure. The correlation matrix shows the correlated movement of the C-alphas in the protein. Displacement and correlation plot shown for (A) C4. (B) TLR1. (C) TLR1/2. (D) TLR4. (E) HLA-A*0201. (F) HLA-B*5301. (G) HLA-CW3. (H) HLA-DRA1. (I) HLA-DRB1. (J) HLA-DP1. (K) HLA-DP2. (L) HLA-DQA1. (M) HLA-DQB1.


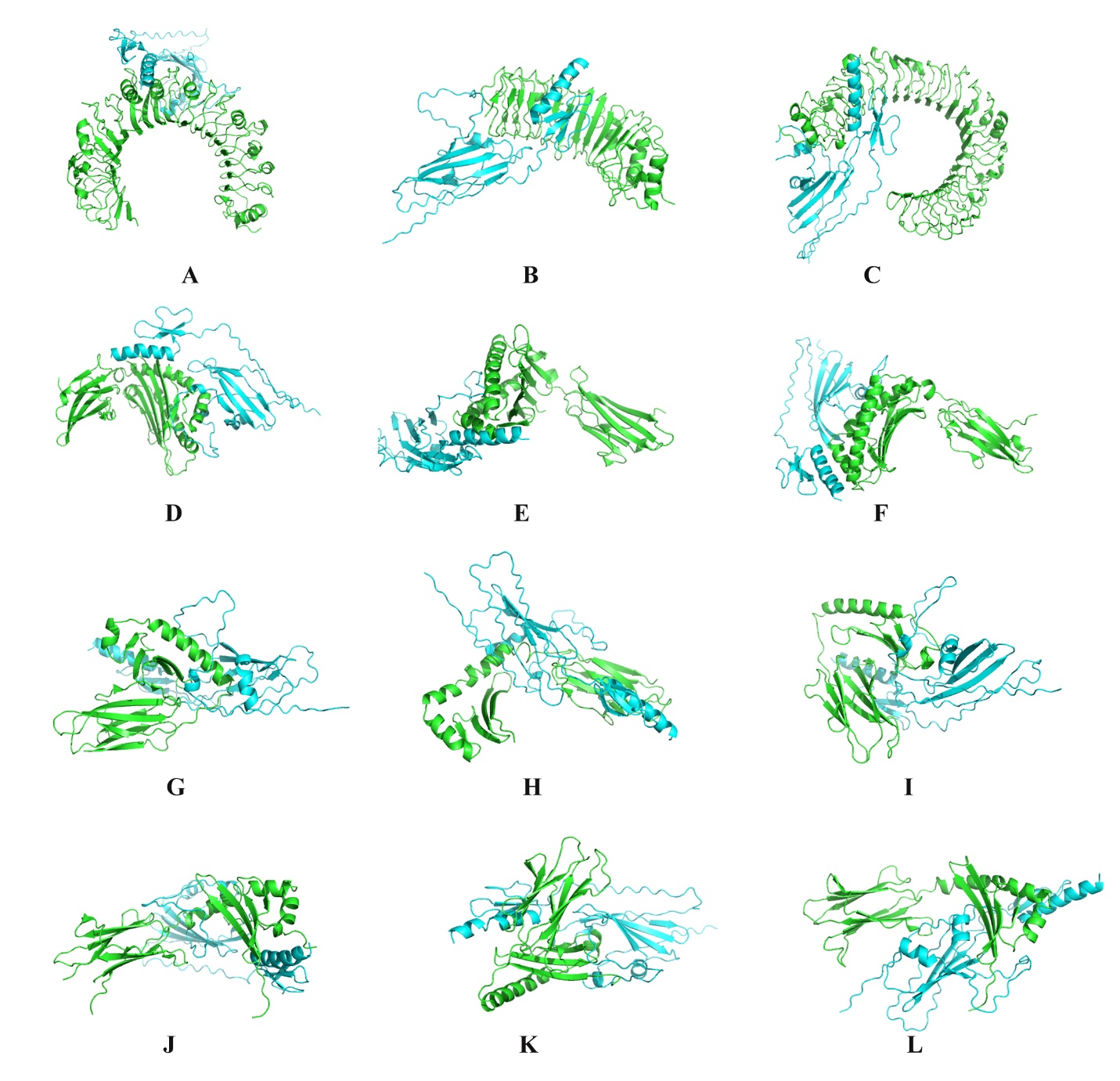


Supplementary Fig. 3. 3D depiction of docked vaccine construct and immune receptors. Vaccine construct is shown in sky blue color. (A) TLR1 and C4 (B) TLR1/2 and C4 (C) TLR4 and C4 (D) HLA-A*0201 and C4 (E) HLA-B*5301 and C4 (F) HLA-CW3 and C4 (G) HLA-DRA1 and C4 (H) HLA-DRB1 and C4 (I) HLA-DP1 and C4 (J) HLA-DP2 and C4 (K) HLA-DQA1 and C4 (L) HLA-DQB1 and C4.


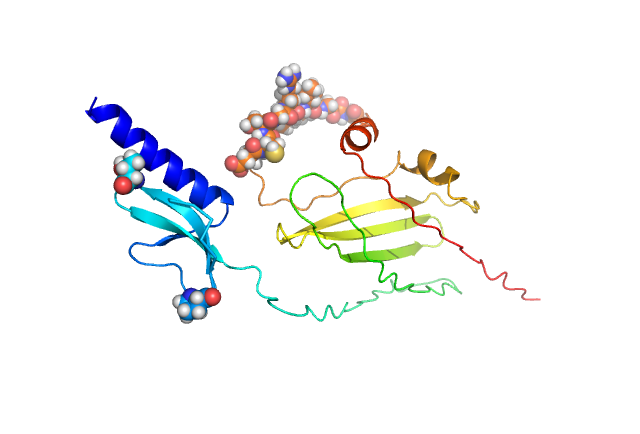


Supplementary Fig. 4. 3D structure of vaccine construct C4 showing residues in sphere with a higher displacement during NMA analysis.

Supplementary Table 1. Designed vaccine constructs using shortlisted epitopes.

| **Serial number (order of epitope)** | **Construct number and sequence** | **Length** | **Antigenicity score** |
| --- | --- | --- | --- |
| 1 (1,2,3) | >C1  EAAAKMAENSNIDDIKAPLLAALGAADLALATVNELITNLRERAEETRRSRVEESRARLTKLQEDLPEQLTELREKFTAEELRKAAEGYLEAATSELVERGEAALERLRSQQSFEEVSARAEGYVDQAVELTQEALGTVASQVEGRAAKLVGIELEAAAKAKFVAAWTLKAAAGGGSNARLGGVNVLGKIYWNGGGGSAKFVAAWTLKAAAGGGSLEASYFNNDYRDLITFGCQIGGGS**HEYGAEALERAG**DQCNYRGNSENYSDCSGRVIKGSGGGS**HEYGAEALERAG**AKFVAAWTLKAAAGGGS | 307 | 0.907 |
| 2 (2,3,1), | >C2  EAAAKMAKLSTDELLDAFKEMTLLELSDFVKKFEETFEVTAAAPVAVAAAGAAPAGAAVEAAEEQSEFDVILEAAGDKKIGVIKVVREIVSGLGLKEAKDLVDGAPKPLLEKVAKEAADEAKAKLEAAGATVTVKEAAAKAKFVAAWTLKAAAGGGSLEASYFNNDYRDLITFGCQIGGGSAKFVAAWTLKAAAGGGSDQCNYRGNSENYSDCSGRVIKGSGGGS**HEYGAEALERAG**NARLGGVNVLGKIYWNGGGGS**HEYGAEALERAG**AKFVAAWTLKAAAGGGS | 287 | 0.824 |
| 3 (3,1,2) | >C3  EAAAKMAENPNIDDLPAPLLAALGAADLALATVNDLIANLRERAEETRAETRTRVEERRARLTKFQEDLPEQFIELRDKFTTEELRKAAEGYLEAATNRYNELVERGEAALQRLRSQTAFEDASARAEGYVDQAVELTQEALGTVASQTRAVGERAAKLVGIELEAAAKAKFVAAWTLKAAAGGGSDQCNYRGNSENYSDCSGRVIKGSGGGSAKFVAAWTLKAAAGGGSNARLGGVNVLGKIYWNGGGGS**HEYGAEALERAG**LEASYFNNDYRDLITFGCQIGGGS**HEYGAEALERAG**AKFVAAWTLKAAAGGGS | 316 | 0.892 |
| **1 (1,2,3)** | **>C4**  **EAAAKGIINTLQKYYCRVRGGRCAVLSCLPKEEQIGKCSTRGRKCCRRKKEAAAKAKFVAAWTLKAAAGGGSNARLGGVNVLGKIYWNGGGGSAKFVAAWTLKAAAGGGSLEASYFNNDYRDLITFGCQIGGGSHEYGAEALERAGDQCNYRGNSENYSDCSGRVIKGSGGGSHEYGAEALERAGAKFVAAWTLKAAAGGGS** | **202** | **1.102** |
| 5 (2,3,1), | >C5  EAAAKMAENSNIDDIKAPLLAALGAADLALATVNELITNLRERAEETRRSRVEESRARLTKLQEDLPEQLTELREKFTAEELRKAAEGYLEAATSELVERGEAALERLRSQQSFEEVSARAEGYVDQAVELTQEALGTVASQVEGRAAKLVGIELEAAAKAKFVAAWTLKAAAGGGSLEASYFNNDYRDLITFGCQIGGGSAKFVAAWTLKAAAGGGSDQCNYRGNSENYSDCSGRVIKGSGGGS**HEYGAEALERAG**NARLGGVNVLGKIYWNGGGGS**HEYGAEALERAG**AKFVAAWTLKAAAGGGS | 307 | 0.889 |
| 6 (3,1,2) | >C6  EAAAK**MAQVINTNSLSLLTQNNLNKSQSSLSSAIERLSSGLRINSAKDDAAGQAIANRFTSNIKGLTQASRNANDGISIAQTTEGALNEINNNLQRVRELSVQATNGTNSDSDLKSIQDEIQQRLEEIDRVSNQTQFNGVKVLSQDNQMKIQVGANDGETITIDLQKIDVKSLGLDGFNV**EAAAKAKFVAAWTLKAAAGGGSDQCNYRGNSENYSDCSGRVIKGSGGGSAKFVAAWTLKAAAGGGSNARLGGVNVLGKIYWNGGGGS**HEYGAEALERAG**LEASYFNNDYRDLITFGCQIGGGS**HEYGAEALERAG**AKFVAAWTLKAAAGGGS | 332 | 0.978 |
| 7 (1,2,3) | >C7  EAAAKMAENPNIDDLPAPLLAALGAADLALATVNDLIANLRERAEETRAETRTRVEERRARLTKFQEDLPEQFIELRDKFTTEELRKAAEGYLEAATNRYNELVERGEAALQRLRSQTAFEDASARAEGYVDQAVELTQEALGTVASQTRAVGERAAKLVGIELEAAAKAKFVAAWTLKAAAGGGSNARLGGVNVLGKIYWNGGGGSAKFVAAWTLKAAAGGGSLEASYFNNDYRDLITFGCQIGGGS**HEYGAEALERAG**DQCNYRGNSENYSDCSGRVIKGSGGGS**HEYGAEALERAG**AKFVAAWTLKAAAGGGS | 316 | 0.895 |
| 8 (2,3,1) | >C8  EAAAKMAENSNIDDIKAPLLAALGAADLALATVNELITNLRERAEETRRSRVEESRARLTKLQEDLPEQLTELREKFTAEELRKAAEGYLEAATSELVERGEAALERLRSQQSFEEVSARAEGYVDQAVELTQEALGTVASQVEGRAAKLVGIELEAAAKAKFVAAWTLKAAAGGGSLEASYFNNDYRDLITFGCQIGGGSDQCNYRGNSENYSDCSGRVIKGSGGGS**HEYGAEALERAG**NARLGGVNVLGKIYWNGGGGS**HEYGAEALERAG**AKFVAAWTLKAAAGGGS | 290 | 0.846 |
| 9 (3,1,2) | >C9  EAAAKGIINTLQKYYCRVRGGRCAVLSCLPKEEQIGKCSTRGRKCCRRKKEAAAKAKFVAAWTLKAAAGGGSDQCNYRGNSENYSDCSGRVIKGSGGGSAKFVAAWTLKAAAGGGSNARLGGVNVLGKIYWNGGGGS**HEYGAEALERAG**LEASYFNNDYRDLITFGCQIGGGS**HEYGAEALERAG**AKFVAAWTLKAAAGGGS | 202 | 1.096 |
